# Macrophage metalloelastase (MMP-12) deficiency does not alter bleomycin-induced pulmonary fibrosis in mice

**DOI:** 10.1186/1476-9255-3-2

**Published:** 2006-02-22

**Authors:** Boris Manoury, Soazig Nenan, Isabelle Guenon, Elisabeth Boichot, Jean-Michel Planquois, Claude P Bertrand, Vincent Lagente

**Affiliations:** 1 INSERM U620, Université de Rennes 1, Rennes, France; 2 Pfizer Global R&D, Fresnes Laboratories, Fresnes, France; 3 EliLilly R&D, Indianapolis, US; 4 AstraZeneca R&D, Alderley Park, Macclesfield, UK

## Abstract

**Background:**

Pulmonary fibrosis is characterized by excessive deposition of extracellular matrix in the interstitium resulting in respiratory failure. The role of remodeling mediators such as metalloproteinases (MMPs) and their inhibitors (TIMPs) in the fibrogenic process remains misunderstood. In particular, macrophage metalloelastase, also identified as MMP-12, is known to be involved in remodeling processes under pathological conditions. However, MMP-12 involvement in pulmonary fibrosis is unknown. Here we investigated fibrotic response to bleomycin in MMP-12 deficient mice.

**Materials and methods:**

C57BL/6 mice, Balb/c mice and MMP-12 -/- mice with a C57BL/6 background received 0.3 mg bleomycin by intranasal administration. 14 days after, mice were anesthetized and underwent either bronchoalveolear lavage (BAL) or lung removal. Collagen deposition in lung tissue was determined by Sircol™ collagen assay, MMP activity in BAL fluid was analyzed by zymography, and other mediators were quantified in BAL fluid by ELISA. Real time PCR was performed to assess gene expression in lung removed one or 14 days after bleomycin administration. Student t test or Mann & Whitney tests were used when appropriate for statistical analysis.

**Results:**

The development of pulmonary fibrosis in "fibrosis prone" (C57BL/6) mice was associated with prominent MMP-12 expression in lung, whereas MMP-12 expression was weak in lung tissue of "fibrosis resistant" (Balb/c) mice. *MMP-12 *mRNA was not detected in MMP-12 -/- mice, in conformity with their genotype. Bleomycin elicited macrophage accumulation in BAL of MMP-12 -/- and wild type (WT) mice, and MMP-12 deficiency had no significant effect on BAL cells composition. Collagen content of lung was increased similarly in MMP-12 -/- and WT mice 14 days after bleomycin administration. Bleomycin elicit a raise of TGF-β protein, MMP-2 and TIMP-1 protein and mRNA in BAL fluids and lung respectively, and no significant difference was observed between MMP-12 -/- and WT mice considering those parameters.

**Conclusion:**

The present study shows that MMP-12 deficiency has no significant effect on bleomycin-induced fibrosis.

## Introduction

Extracellular matrix (ECM) remodelling is a key factor of numerous interstitial lung diseases. Airway remodelling-associated disorders of ECM can be illustrated by different pathological situations including emphysema and pulmonary fibrosis. In the first, progressive proteolytic ECM degradation is prevailing, whereas in the latter excessive matrix deposition occurs. Both phenomena are hypothesized to result partially from an imbalance of ECM homeostasis and protease – antiprotease activity [1,2] which is partially regulated by potent fibrogenic growth factors such as TGF-β [3,4].

Metalloproteinases (MMPs) are a family of zinc-binding endopeptidases which proteolytic activity is involved in normal and pathological ECM turnover. MMPs activity is regulated by their natural tissue inhibitors, TIMPs. MMPs have been implicated in lung pathological conditions, including fibrosis [5], emphysema [1] and asthma [6]. Matrilysin (MMP-7) was shown to have great importance in idiopathic pulmonary fibrosis and found to be dramatically involved in bleomycin-induced pulmonary fibrosis in mice [7]. Also, gelatinase-A (MMP-2) seems to be a good marker of tissue remodelling. It is localized in the area of fibroproliferation and basal membrane disruption [8] and shows intense activity in experimental models of pulmonary fibrosis [9-11]. Hence, TIMP-1 has been increasingly associated with pulmonary fibrosis [8,9,12,13]. It has been suggested that abnormal alteration of MMPs/TIMPs balance could lead to disruption of lung tissue, and/or accumulation of extracellular matrix without adequate repair, leading to impairment of lung function [2,3,8].

Macrophage metalloelastase, also identified as MMP-12, has been previously described as a key factor of pathological progressive proteolytic destruction of ECM. Indeed, MMP-12 has been reported to be essential in tissue remodelling associated with emphysema in mice exposed to cigarette smoke [14]. In addition, an increased expression of MMP-12 in macrophages from patients with COPD was recently reported [15]. MMP-12 has potent ECM remodelling properties due to its specific elastolytic activity, but may also participate to the inflammatory process through the activation of TNF-alpha [16]. Moreover, MMP-12 presents potent direct pro-inflammatory properties including the ability to induce neutrophil influx, cytokine and chemokine production [17]. MMP-12 seems to be involved in numerous models of acute lung inflammation [16,18-20]. Although the role of MMP-12 in animal models of emphysema is well documented, its involvement in pulmonary fibrosis remains unclear. We therefore investigated the involvement of MMP-12 in the development of bleomycin-induced pulmonary fibrosis. Firstly, we investigated differential MMP-12 expression in lungs of "fibrosis prone" (C57BL/6) mice and in "fibrosis resistant" (Balb/c) mice [12,21] after bleomycin administration. Secondly, we compared the inflammatory and fibrotic responses of MMP-12 null mice with those of their wild type C57BL/6 littermates.

## Materials and methods

### Materials

Seven-week-old Balb/c and C57BL/6 male mice were purchased from CERJ (Le Genest Saint Isle, France) and quarantined for 1 week before experiments. MMP-12 -/- mice were obtained from Charles River laboratories following a transfer from Washington University [22] and rederivation on C57BL/6 background. C57BL/6 mice were used as wild type (WT) control mice. They were housed under controlled and ethical conditions that complied with the Interdisciplinary Principles and Guidelines for the Use of Animals in Research, Marketing and Education, New York Academy of Sciences' Ad Hoc Committee on Animal Research.

The following materials were used: bleomycin sulfate from Bellon Laboratories (Montrouge, France); gelatin, Triton X-100, Coomassie Brilliant Blue, Tween 20 solution, and trypan blue from Sigma (St Louis, MO, USA) ; pepsin was from Fluka (Buchs, Switzerland) ; May-Grünwald and Giemsa stains from RAL (Paris, France); sodium pentobarbital from Sanofi Santé Animale (Libourne, France); etomidate (Hypnomidate®, 2 mg/mL) from Janssen-Cilag (Issy-les-Moulineaux, France); acrylamide, sodium dodecyl sulfate (SDS), Tris, and BSA from Eurobio (Les Ulis, France); ELISA kits for TGF-β and TIMP-1 detection were from R&D Systems (Minneapolis, MN, USA); isopentane from Prolabo (Fontenay-sous-Bois, France); a low-range weight marker for SDS-PAGE from Biorad (Munich, Germany).

### Bleomycin administration

Pulmonary fibrosis was induced by intranasal (i.n.) instillation, as previously described [5,22]. Each mouse was administered 0.3 mg bleomycin dissolved in 0.9% NaCl solution. Control mice received saline vehicle only. One day or 14 days after i.n. administration, mice were quickly anesthetized by an i.p. injection of sodium pentobarbital (60 mg/kg) and underwent either bronchoalveolar lavage (BAL) or lung removal. These samples were stored at -80°C until further analysis.

### Bronchoalveolar lavage and preparation of tissue homogenates

Mice were anesthetized with an i.p. administration (20 mL/kg) of sodium pentobarbital 0.6%. Bronchoalveolar lavage (BAL) fluids were obtained by washing the airways 10 times with 0.5 mL of 0.9% NaCl solution at 37°C with a 1 mL syringe. The BAL fluid was centrifugated (600 g for 10 min, 4°C), and the supernatant of the first two fractions (1 mL) divided into aliquots and frozen at -80°C until analysis. The cell pellets were then pooled with the last fractions. Total cells were counted with a Coulter Z2^® ^(particle counter and size distribution analyzer, Beckman Coulter). Red blood cells were eliminated by adding 3 mL of distilled water for 30 seconds and then 1 mL of KCl 0.6 M onto the pellets. After centrifugation (600 g for 10 min, 4°C), supernatant was eliminated and the cells were suspended in 1 mL of PBS. They were then cytospun at 700 rpm for 10 minutes (Cytospin 3^®^, Thermo Shandon, Ltd, Astmoor, United Kingdom) and stained with the May-Grünwald Giemsa method. Differential cell counts of 200 cells used standard morphological criteria.

### Zymographic analysis of MMPs

Since some MMPs can degrade gelatin, zymographic techniques were used to detect MMPs in BAL. In non reducing conditions and in the presence of SDS, as previously described [23], aliquots of BAL fluid or lung homogenate underwent electrophoresis onto a 6% acrylamide stacking gel /10% acrylamide separating gel containing 1 mg/mL gelatin. After electrophoresis, gels were washed twice with 2.5% Triton X-100, rinsed with water, and incubated overnight at 37°C in 50 mM Tris, 5 mM CaCl_2_, 2 μM ZnCl2, pH = 8. The gels were stained with Coomassie Brilliant Blue in a solution of 25% ethanol-10% acetic acid in water and rinsed in an identical solution. Gelatinase activity appeared as clear bands against blue background. We used recombinant protein molecular weight markers (20 kDa-214 kDa) to estimate the molecular weights of the gelatinolytic bands. Relative enzyme amounts were quantified by measuring the intensity of the bands with a densitometric analyzer (Bio-Profile, Vilber-Lourmat, Marne la Vallée, France). Results were expressed as a percentage of the band of migration of one control BAL sample loaded onto each gel. This sample was used as an internal standard to allow further comparisons between gels.

### Determination of TGF-β-1 and TIMP-1 in BAL

The amounts of total TGF-β-1 and TIMP-1 were determined with ELISA methods, performed according to the manufacturer's recommendations. Assay sensitivity was 15 pg/mL for TGF-β-1 and 31 pg/mL for TIMP-1. As ELISA protocol called for processing samples in 0.6N HCl before the assay, both TGF-β-1 latent and active forms were measured at the same time.

### In vivo expression of α1(I) collagen, Mmp-12, Mmp-2, and Timp-1 mRNA in lungs

For quantification of α*1(I) collagen, Mmp-12, Mmp-2 *gene expressions in lung tissue, mice were sacrificed fourteen days after either bleomycin or saline administration. Frozen lung samples were ground to a fine powder using a mortar and a pestle, and homogenized in 2 mL of Trizol reagent (Invitrogen Life technology, Paisley, UK). After vigorous shaking, chloroform was added and the samples were centrifuged at 12 000 g for 20 min. Total RNA was precipitated with isopropanol, and dissolved in RNAse-free water. RNAs were reversed-transcribed into cDNA using SuperScript™ II (Invitrogen Life technologies, Paisley, UK), following the manufacturer's protocols with the following exceptions : 1 μg of total RNA and random hexamer primers (500 ng, Promega) were used in each RT reaction. cDNA were stored at -20°C until use as template in subsequent polymerase chain reaction. Real-time quantitative PCR was performed by the fluorescent dye SYBR Green methodology, using SYBR Green PCR Master Mix (Applied Biosystems), and the ABI Prism 7000 apparatus (Perkin-Elmer, Foster city, CA, USA). Primers pairs for PCR reaction were chosen with the Primers 3 software [24], excepted for TIMP-1 which were already described in literature [12] (Table 1). Briefly, cDNA were mixed with 8 μL SYBR Green Master Mix, 300 nM of each primer, in a final volume of 16 μL. A first step of 10 min at 95°C was followed by 40 cycles of amplification (95°C for 15 sec and 60°C for 60 sec). Water and RNA that had not been subjected to the RT step were used as negative control. For each sample, the ABI Prism 7000 software provided an amplification curve constructed by relating the fluorescence signal intensity (normalized to the fluorescence of ROX internal passive reference) to the cycle number. The relative quantification of the steady-state of the target mRNA levels was calculated after normalization of the total amount of cDNA tested by an active reference, *18s*. Fluorescence data from each sample were analyzed with the 2^[-ΔΔ*Ct*] ^method with the mean of the saline treated control group as the calibrator : relative copy number of GI RNA = 2^[-ΔΔ*Ct*]^, were ΔΔCt = [Ct _(GI) _(Unknown sample) – Ct _18s _(Unknown sample)] – [(Ct _(GI)_(calibrator) – Ct _18s _(calibrator)], GI is the gene of interest, and Ct, the cycle threshold that was defined as the cycle number at which a significant increase in the fluorescence signal crosses an arbitrary intensity threshold.

**Table 1:** Genes and primers for real time PCR.

**Gene**	**Sense Primer (5' to 3')**	**Reverse Primer (5' to 3')**
*18S*	CGCCGCTAGAGGTGAAATT	TTGGCAAATGCTTTCGCTC
α*1(I) collagen*	TCCTGCTGGTGAGAAAGGAT	TCCAGCAATACCCTGAGGTC
*Timp-1*	GTGGGAAATGCCGCAGAT	GGGCATATCCACAGAGGCTTT
*Mmp-2*	CCAGATACCTGCACCACCTT	GTTGAAGGAAACGAGCGAAG
*Mmp-12*	GCTAGAAGCAACTGGGCAAC	ACCGCTTCATCCATCTTGAC

### Collagen measurement

For quantification of collagen in lung tissue, mice were sacrificed fourteen days after BLM administration. Lungs were freshly removed and frozen in isopentane using liquid nitrogen. Frozen lungs were ground to a fine powder with a mortar and pestle, weighed, and homogenized in acetic acid 0.5 M containing pepsin (2.7 U/mL) pH = 7.4. Total soluble collagens were extracted overnight at room temperature by using 5 mg/ml pepsin (2.7 U/mL) in 0.5 M acetic acid and measured by the Sircol™ collagen assay (Biocolor Ltd, Newtonabbey, UK).

### Expression of the results and statistical analysis

Results were expressed as means ± SEM. Normality of data distribution was determined using the Kolmogorov-Smirnov normality test. The groups were compared according to treatment and strain by using either student t test or nonparametric Mann-Withney Rank Sum Test, when data distribution was respectively normal or not. For each analysis, P values under 0.05 were considered to be statistically significant, unless another range is specified.

## Results

### In vivo expression of MMP-12 mRNA in lung tissue of Balb/c and C57BL/6 mice

**Figure 1:**
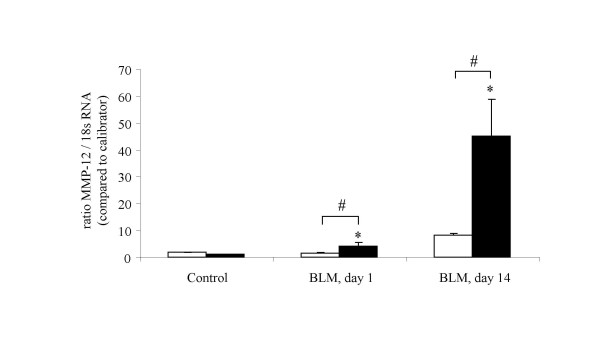
*MMP-12 *mRNA analysis by real time quantitative polymerase chain reaction (PCR) in lung of Balb/c (blank bars) and C57BL/6 mice (solid bars), removed one day or 14 days after intranasal administration of bleomycin (BLM) or vehicle saline (Control). Results are presented as mean ± SEM of the ratio of the number RNA copies in the unknown sample in comparison to the calibrator. *: P < 0.05 compared to corresponding control. N = 3–5.

*MMP-12 *mRNA expression in lung tissue was firstly investigated in Balb/c and C57BL/6 mice (figure 1). One day after bleomycin administration, *MMP-12 *mRNA level was increased in C57BL/6 mice (4 fold), but not in Balb/c mice. At day_14_, bleomycin elicited a raise of *MMP-12 *mRNA level both in Balb/c and in C57BL/6 mice. However, the *MMP-12 *mRNA level was strikingly increased in C57BL/6 in comparison to Balb/c mice (43 fold increase versus 4.7, respectively, P < 0.05).

### In vivo expression of MMP-12 mRNA in lung tissue of MMP-12 -/- and WT mice

*MMP-12 *mRNA was not detected in lung of MMP-12 -/- mice, confirming the genotype of the mice used for the study. In contrast, MMP-12 was detected in control WT mice, and was increased after bleomycin administration (50 fold increase).

### Cell composition of bronchoalveolar lavage (BAL) fluids of MMP-12 -/- and WT mice

The total cell number as well as the absolute number of different cell types recovered in the BAL fluids of mice from each experimental group is reported in table 2. Bleomycin administration elicited a significant increase in the total cell count in the BAL fluids in both WT mice and in MMP-12 -/- mice. This was due to a large increase in the number of alveolar macrophage and, in a lesser extent, to a rise in the number of neutrophils. Eosinophils and lymphocytes count raised only in BAL of WT mice after bleomycin administration. Although the leucocytes influx seems to be less important in BAL of MMP-12 -/-mice than in WT mice, no significant difference between both strains could be observed.

**Table 2:** Total and differential cell counts of BAL fluid from MMP-12 -/- and WT mice 14 days after intranasal administration of bleomycin (BLM) or saline vehicle (Control). Results are presented as the mean (.10^3 ^cells) ± SEM. n: number of mice. a: P < 0.05, b: P < 0.01, c: P < 0.001 compared with control mice exposed to saline solution only.

Treatment	Strain	N	Total cells	Macrophages	Neutrophils	Eosinophils	Lymphoytes
Control	WT	5	579 ± 92	561 ± 79	13 ± 10	0 ± 0	6 ± 6
	MMP-12 -/-	5	547 ± 92	540 ± 94	2 ± 2	0 ± 0	4 ± 4
BLM	WT	8	2204 ± 284^c^	1783 ± 210^c^	285 ± 78^b^	55 ± 19^b^	81 ± 33^b^
	MMP-12 -/-	8	1352 ± 355^a^	1152 ± 255^a^	104 ± 62^b^	19 ± 17	76 ± 55

### Quantification of collagen deposition in lung tissue of MMP-12 -/- and WT mice

**Figure 2:**
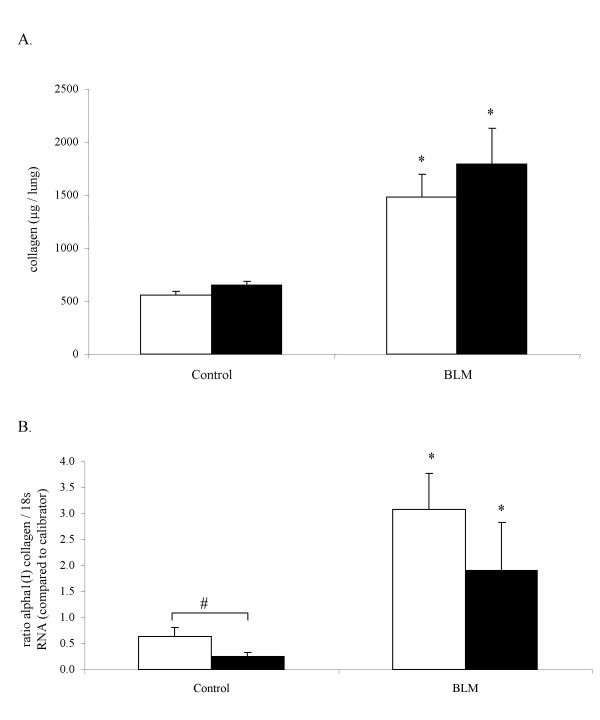
A : Collagen content (mg/lung) in lung from WT (blank bars) and MMP-12 -/- mice (solid bars), 14 days after intranasal administration of bleomycin (BLM) or saline vehicle (Control). B : α*1 (I) collagen I *mRNA analysis by real time quantitative polymerase chain reaction (PCR) amplification. Results are presented as mean ± SEM. *: P < 0.05 compared to corresponding control. #: P < 0.05 compared to WT BLM. N = 4–9.

Collagen content of lung tissue was determined by the Sircol™ collagen assay following extraction of soluble collagen from lung removed at day 14. Indeed, bleomycin-induced collagen deposition and tissue fibrosis was previously shown to be effective at this stage, under similar conditions [5,7,10]. Bleomycin elicited an increase of collagen content in both WT and MMP-12 -/- mice, in a similar extent in both groups (figure 2A). Similarly, *α1(I) collagen *mRNA was induced both in WT and MMP-12 -/- mice (figure 2B). Basal mRNA level was higher in control WT mice than in control MMP-12 -/- mice, whereas it was similar in mice that were received bleomycin in both groups.

### TGF-β and TIMP-1 levels in BAL fluids of MMP-12 -/- and WT mice

**Figure 3:**
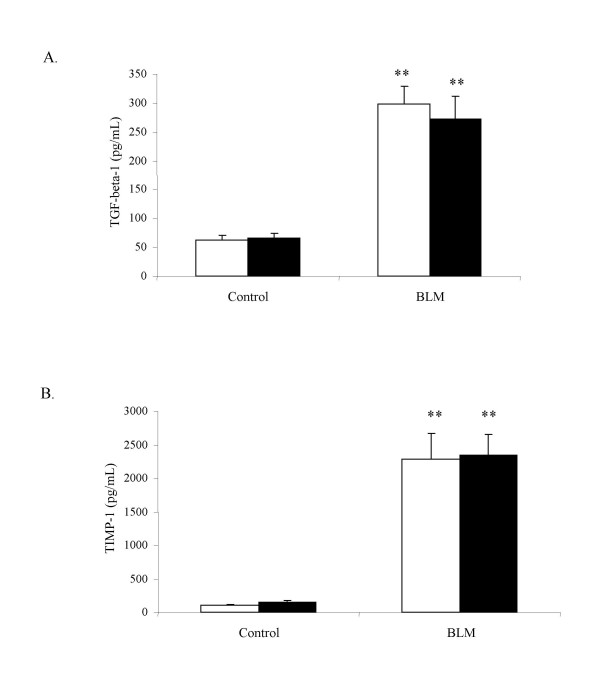
Levels of TGF-β-1 (A), and TIMP-1 (B) in BAL supernatant fluids, recovered from WT (blank bars) and MMP-12 -/- mice (solid bars), at day_14 _after intranasal administration of bleomycin (BLM) or saline vehicle (Control). For TGF-β-1 and TIMP-1, results are represented as the mean (pg/ml) ± SEM. ** : P < 0.01 compared to corresponding control. n = 5–8.

Bleomycin elicited a significant increase of TGF-β-1 protein in BAL fluids of WT and in MMP-12 -/- mice (figure 3A), in a similar extent in both groups. TIMP-1 protein level showed a similar profile, as it was increased 14 days after bleomycin administration in both WT and MMP-12 -/- mice (figure 3B). No difference between WT and MMP-12 -/- was noted.

### Metalloproteinase activity in BAL fluids of of MMP-12 -/- and WT mice

**Figure 4:**
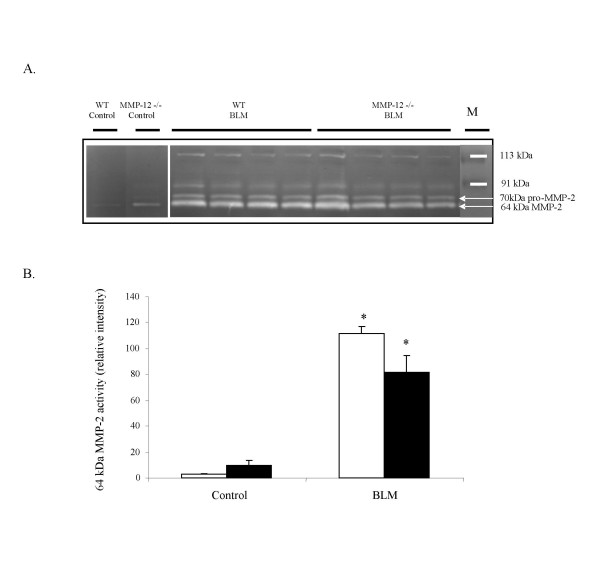
A. Representative gelatin zymogram of BAL supernatant fluids 14 days after intranasal administration of bleomycin (BLM) or saline vehicle (Control) to WT and MMP-12 -/- mice. M: molecular weight marker. B : Quantification by densitometry of 64 kDa MMP-2 gelatinase activity on zymograms of BAL fluid, performed 14 days after intranasal administration of bleomycin (BLM) or vehicle saline (Control), in wild type mice (WT) (blank bars) and MMP-12 -/- mice (solid bars). Results are represented as the mean ± SEM. * : P < 0.05 compared to corresponding control. N = 4–8.

By zymography, identification of gelatinases was based on substrate specificity and molecular weight (figure 4A). Zymograms loaded with BAL fluid samples exhibited the following gelatinolytic bands : pro-MMP-2 (70 kDa), and MMP-2 (64 kDa). These gelatinolytic activities were inhibited by EDTA 10 mM, indicating that corresponding enzymes belong to MMPs family (data not shown). Molecular weights of latent and active forms of murine MMP-2 were similar to those described in literature [25,26]. Administration of bleomycin induced an increase of both latent and active forms of MMP-2 (Fig. 4A). Active 64 kDa form of MMP-2 was measured (figure 4B). No significant difference between WT and MMP-12 -/- groups was observed.

### Mmp-2 and Timp-1 gene mRNA analysis by real time PCR in MMP-12 -/- and WT mice

**Figure 5:**
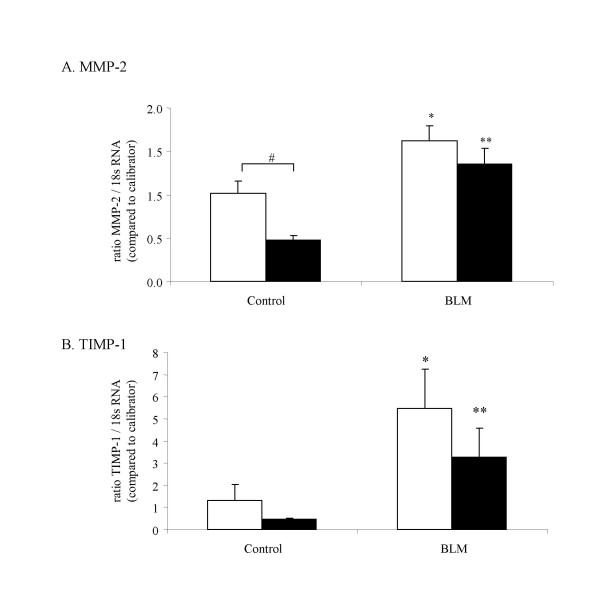
*Mmp-2 *(A), and *Timp-1 *(B) mRNA analysis by real time polymerase chain reaction (PCR) amplification in lung from WT (blank bars) and MMP-12 -/- mice (solid bars) at day_14 _after intranasal administration of bleomycin (BLM) or saline vehicle (Control). Results are presented as mean ± SEM. *: P < 0.05 compared to corresponding control. #: P < 0.05 compared to WT Control. N = 4–9.

Effects of bleomycin administration on respective mRNA level of *Mmp-2 *gene and *Timp-1 *gene expressions were investigated in lung by real time PCR method (figure 5).

Bleomycin induced *Mmp-2 *mRNA expression in both WT and MMP-12 -/- mice (respectively 1.6 fold and 2.8 fold) (figure 5A). Basal level was higher in WT mice compared to MMP-12 -/-.

*Timp-1 *mRNA was induced by bleomycin in MMP-12 -/- (7.3 fold), and WT (4.1 fold), (figure 5B).

## Discussion

The present study showed that MMP-12 -/- mice developed pulmonary fibrosis in a similar intensity to WT mice and the level of TIMP-1 protein and *Timp-1 *mRNA is similar in both strains.

Metalloproteinases have been described to be involved in the remodelling process and ECM turn over in several respiratory diseases associated with inflammation, including pulmonary fibrosis [27]. Indeed, it has been previously reported that a broad range MMP inhibitor, batimastat, prevented bleomycin-induced pulmonary fibrosis in mice [5]. In addition, several studies focussing on specific members of MMP family have been providing information about their relative importance in the process. In particular, the involvement of gelatinases (MMP-2 and MMP-9) has been described in human and in *in vivo *experimental animal models [5,23]. However, MMP-9 null mice were not protected from extensive collagen deposition following bleomycin administration, but did not develop small airway fibrosis [28].

MMP-12, also known as macrophage metalloelastase, and neutrophil elastase (NE) are two major elastases involved in lung inflammation and remodelling. Moreover, NE null mice have already been described to be resistant to bleomycin-induced pulmonary fibrosis [29]. In contrast, the role of MMP-12 in the development of fibrosis is relatively unknown.

In a first set of experiments, we investigated *MMP-12 *mRNA level in lung of Balb/c and C57BL/6 after bleomycin administration. Indeed, C57BL/6 mice are known to be "fibrosis prone" strain, whereas Balb/c mice are described as resistant to the development of pulmonary fibrosis [12,21]. In conformity with this, the preliminary hydroxyproline level measurement revealed that C57BL/6 mice developed important collagen accumulation 14 days after 0.1 mg bleomycin administration, whereas no raise of collagen level was observed in lung of Balb/c mice [9]. Here, we show that *MMP-12 *mRNA level in lung tissue was increased in C57BL/6 mice at day_1 _and day_14_, whereas a slight raise of *MMP-12 *mRNA was detected only at day_14 _in Balb/c. We confirm here that bleomycin elicits *MMP-12 *mRNA induction in lung tissue of WT mice, as previously described by Swiderski et al. [27]. Moreover, as *MMP-12 *mRNA level is associated with strain-dependent susceptibility to develop bleomycin-induced pulmonary fibrosis, MMP-12 seems to be a relevant candidate for the determination of a pivotal proteolytic element in the development of pulmonary fibrosis. Hence, we investigated the effect of MMP-12 deficiency in bleomycin-induced fibrosis model which hitherto has not been related.

In the present study, we observed that MMP-12 -/- mice responded to bleomycin administration by an increase of collagen content in lung tissue, which is the hallmark of pulmonary fibrosis. This raise did not differ significantly from observations in WT mice, suggesting that MMP-12 is not necessary for the development of bleomycin-induced pulmonary fibrosis. Similarly, Lanone et al. [18] observed in IL-13-induced injury in mice that MMP-12 deficiency did not alter subepithelial fibrosis following inducible IL-13 transgene expression. Consistent with their genotype, MMP-12 -/- mice did not demonstrate MMP-12 expression.

It is interesting to observe that all mRNA level in MMP-12 -/- mice are lower than in wild type under basal conditions, although all mRNA were all extracted and reverse transcribed during the same run. However, such differences are not observed at the protein level regarding either collagen, MMP-2 nor TIMP-1, suggesting that a strong post-traductional regulation of gene expression occurs.

MMP-12 is known to be a prominent protease produced by activated alveolar macrophage [1,30]. We observed a marked accumulation of macrophages in BAL of WT mice 14 days after bleomycin administration. Taken together, these data could mean than bleomycin induces infiltration and activation of macrophages in respiratory tract, alveolar spaces and interstitium, leading to prominent expression of MMP-12 in lung. Therefore MMP-12 does not seem to be implicated in the fibrosis genesis, but may be an indicator of macrophage activation and recruitment following bleomycin administration.

In contrast, several studies reported that MMP-12 was necessary for accumulation of macrophages in the airways, and associated with alveolar enlargement and emphysematous lesions after cigarette smoke exposure [14] or inducible IL-13 transgene expression [18]. Hence, Nénan et al. [17] recently demonstrated that instillation of recombinant catalytic domain of human MMP-12 to mice induced inflammation with important and stable macrophage recruitment in airways. Parks and Shapiro [30] suggested that MMP-12 may actually be involved in leucocyte recruitment and activation. Indeed, it has been shown that proteolytically generated elastin fragments mediate monocyte chemotaxis [31]. However, in our model, BAL total cell count and macrophage accumulation were not significantly altered by MMP-12 deficiency at day_14_. This may suggest that, at that time point, macrophage accumulation is monitored by other mediators than MMP-12.

Taken together these results suggest that bleomycin-induced fibrosis and associated inflammation involve different mechanisms from MMP-12 dependent pathways. Several hypothesis suggest that crucial components of fibrogenic process are due to remodelling disorders involving growth factors such as TGF-β [2] and a non degrading microenvironment created by a "shield" of protease inhibitors, including TIMP-1 [2,8]. TGF-β has been demonstrated to be a prominent fibrogenic mediator in many organs, including lung [32,33]. Moreover, TGF-β presents a pivotal situation by regulating global lung tissue remodelling. Indeed, mice lacking integrin αvβ6 (integrin αvβ6 null mice) fail to activate TGF-β and develop an age-related emphysema, which is MMP-12-dependent [4]. MMP-12 in lung has been described to be down regulated by TGF-β signalling pathway [3,4]. In our experimental model of pulmonary fibrosis, TGF-β-1 protein was induced similarly in both MMP-12 -/- and WT mice. In accordance with our results, Lanone et al. [18] did not report alteration of total TGF-β in BAL fluid of MMP-12 -/- mice after IL-13 transgene expression. Interestingly, in WT mice, bleomycin elicits both MMP-12 and TGF-β increase. Therefore further investigations are required in order to explain why MMP-12 expression coexists with high TGF-β level.

Growing evidence tends to establish TIMP-1 as a major fibrogenic effector in lung. Indeed, upon fibrogenic stimuli, a large amount of TIMP-1 in the remodelling tissue is supposed to participate to the creation of a non degrading environment, leading to alteration of protease/anti-protease balance, extracellular matrix accumulation, and tissue fibrosis [2,8]. Moreover, TIMP-1 has been designated as a key factor to explain strain-dependent susceptibility to develop fibrogenic response to transfer of active TGF-β in lung of mice [12]. We previously observed that TIMP-1 expression and protein were importantly increased by bleomycin administration. We and others had previously reported such an induction in lung of C57BL/6 mice after bleomycin administration [5,10,13,27]. According to our results, MMP-12 deficiency does not seem to be involved in TIMP-1 regulation and this would be consistent with the development of pulmonary fibrosis in MMP-12 -/- mice.

Gelatinases MMP-9 and MMP-2 have been observed in various lung injury models [10,18,27], and in pulmonary fibroses in humans [8,34]. Studying the present study, we detected pro-MMP-2 and MMP-2 activities in BAL. Here, MMP-2 protein and mRNA levels were both increased at day_14 _after bleomycin administration, in a similar extent in MMP-12 -/- and WT mice. This result does not appear consistent with the hypothesis that IL-13-induced MMP-2 increase was mediated at least partially by a MMP-12-dependent pathway [18]. Therefore, even though Th2 cytokine IL-13 has been involved in bleomycin-induced pulmonary fibrosis [35], IL-13-induced injury model seems to follow different pathways, including different participation of MMP-12. Moreover, IL-13-induced injury is associated with airspace enlargement, which is not observed in bleomycin-induced lung injury.

## Conclusion

In conclusion, we demonstrated that MMP-12 does not appear to be involved in the fibrogenic pathway of bleomycin-induced lung injury. MMP-12 deficiency did not influence the bleomycin-induced raise of neither TGF-beta-1 nor TIMP-1 in lung, which are described as important pro-fibrogenic effectors. Moreover, in this model, MMP-12 deficiency has no influence on macrophages accumulation in lung. Accordingly to the protease/antiprotease imbalance hypothesis, accumulation of extracellular matrix may be due by dysfunction of other proteases than MMP-12.

## Competing interests

The author(s) declare that they have no competing interests.

## Authors' contributions

BM directed the study, experimental design. Experimental procedures were performed by BM, SN, IG, EB and VL. VL, EB, JMP and CPB supervised experimental design and procedures. VL and EB supervised the manuscript, revising it critically for important intellectual content. All authors have given final approval of the version to be published.
